# Liquid Crystal Polymer-Based Miniaturized Fully Implantable Deep Brain Stimulator

**DOI:** 10.3390/polym15224439

**Published:** 2023-11-16

**Authors:** Seung-Hee Ahn, Chin Su Koh, Minkyung Park, Sang Beom Jun, Jin Woo Chang, Sung June Kim, Hyun Ho Jung, Joonsoo Jeong

**Affiliations:** 1 Department of Electrical and Computer Engineering, College of Engineering, Seoul National University, Seoul 08826, Republic of Korea; 2 Department of Neurosurgery, Yonsei University College of Medicine, Seoul 03722, Republic of Korea; 3 Department of Electronic and Electrical Engineering, Ewha Womans University, Seoul 03760, Republic of Korea; 4 School of Biomedical Convergence Engineering, Pusan National University, Yangsan 50612, Republic of Korea

**Keywords:** liquid crystal polymers, deep brain stimulation, implantable electronics, von Frey test

## Abstract

A significant challenge in improving the deep brain stimulation (DBS) system is the miniaturization of the device, aiming to integrate both the stimulator and the electrode into a compact unit with a wireless charging capability to reduce invasiveness. We present a miniaturized, fully implantable, and battery-free DBS system designed for rats, using a liquid crystal polymer (LCP), a biocompatible and long-term reliable material. The system integrates the simulator circuit, the receiver coil, and a 20 mm long depth-type microelectrode array in a dome-shaped LCP package that is 13 mm in diameter and 5 mm in height. Wireless powering and control via an inductive link enable device miniaturization, allowing for full implantation and, thus, the free behavior of untethered animals. The eight-channel stimulation electrode array was microfabricated on an LCP substrate to form a multilayered system substrate, which was monolithically encapsulated by a domed LCP lid using a specialized spot-welding process. The device functionality was validated via an in vivo animal experiment using a neuropathic pain model in rats. This experiment demonstrated an increase in the mechanical withdrawal threshold of the rats with microelectrical stimulation delivered using the fully implanted device, highlighting the effectiveness of the system.

## 1. Introduction

Deep brain stimulation (DBS) is a technique that uses electrical pulses to modulate the activity of specific brain regions associated with motor, sensory, and cognitive functions, such as the thalamus and basal ganglia [1,2,3,4,5]. Since 1997, DBS has been used to treat neurological and psychiatric disorders in more than 150,000 patients [6,7,8]. The subthalamic nucleus and the internal globus pallidus are common targets in the DBS treatment of Parkinson’s disease. Several studies have shown that DBS potentially improves cognitive function in Alzheimer’s disease, obsessive–compulsive disorder, and depression [9,10,11].

From a device perspective, a conventional DBS system consists of three components: an electrode that stimulates the targeted brain region, an implantable pulse generator (IPG) that generates stimulation pulses, and a lead wire that connects the IPG and the electrode [12]. For example, Medtronic 3389 is a commercially available DBS electrode with a cylindrical structure that is 1.27 mm in diameter and has four 1.5 mm wide ring-shaped platinum contacts spaced 0.5 mm apart [13]. The electrodes are located near the tip of the shank and configured to deliver bipolar stimulation [14]. The IPG generates electrical stimulation pulses with programmable pulse parameters, such as current amplitudes, pulse rates, and pulse widths [15]. Primary batteries powered early IPGs; therefore, the batteries had to be surgically replaced approximately every 10 years after implantation. While recent rechargeable IPGs have eliminated the need for such battery replacement, their bulky size (approximately 50 mm) and weight (approximately 30–50 g) still pose a challenge when they are being implanted near the brain. Instead, these IPGs are usually implanted below the collarbone in the chest and require long lead wires to pass under the skin to connect to the electrode hole in the skull, which increases the surgical complexity and the risks of infection and mechanical failure.

Therefore, a significant obstacle in advancing DBS technology is device miniaturization to minimize invasiveness. Although the idea of miniaturized DBS systems integrating the IPG and the electrode into a compact device has been conceptually suggested [16,17], there is still a gap in translating this concept into an actual stem-level implementation and in vivo validation of such head-mountable DBS devices. Miniaturization becomes more challenging when intended for animal experiments, which is a critical step in evaluating new therapeutic technologies and conducting basic neuroscience research to understand brain functions. Existing DBSs for small animals typically rely on a percutaneous system with a wired stimulator [18,19,20,21,22]. A miniaturized, fully implantable DBS system that allows the untethered small animals to freely move around during electrical stimulation would lead to a series of possibilities in the field of neural engineering.

Conventionally implanted biomedical devices, such as DBSs, rely on metallic packaging. Despite their hermeticity, metallic packages are bulky, heavy, and difficult to miniaturize. Although polymeric devices have been proposed, their long-term reliability remains to be proven and is primarily limited by the moisture-absorbing nature of polymers. However, liquid crystal polymers (LCPs) have gained increasing interest for their low moisture absorption rates (<0.04%) compared to those of conventional biocompatible polymers, such as polyimide (~2.8%), parylene-C (0.06–0.6%), and silicone elastomers [23]. This makes LCP attractive as a substrate and a packaging material for implantable neural stimulators as it can potentially provide enhanced long-term reliability under aqueous conditions [23,24,25,26]. The chemically inert nature and mechanical robustness of LCP also make it compatible with standard microfabrication processes. In addition, the thermoplasticity of LCP allows the formation of various 3D structures via heat pressing.

In this study, we present an LCP-based miniaturized head-mounted DBS. The device comprises an eight-channel microelectrode array and a stimulation circuit integrated into a compact LCP package 13 mm in diameter that is wirelessly powered via an inductive link. The microelectrode array was fabricated on an LCP film, constituting a multilayered system substrate laminated via thermal pressing. A finite element method (FEM) simulation was conducted to verify the radiation safety of the device upon wireless powering. An in vivo experiment on a neuropathic pain model in rats verified the device’s efficacy.

## 2. Materials and Methods

### 2.1. System Overview

**Figure 1:**
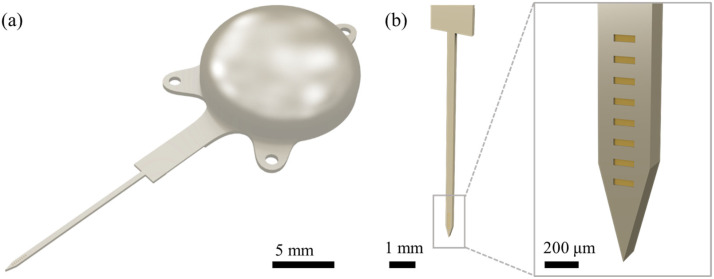
Concept images of the LCP-based miniaturized head-mountable DBS system. (**a**) Three-dimensional model of the LCP-based monolithic DBS with a dome-shaped package and (**b**) eight-channel microelectrode array.

The proposed LCP-based fully implantable wireless DBS system in Figure 1a comprises an electrode array, a stimulation-generating circuit, and a package. The eight-channel electrode array microfabricated on an LCP substrate delivers current stimulation pulses to the targeted tissue, while the stimulator circuit on a printed circuit board (PCB) generates stimulation pulses with the desired parameters and is powered and controlled using an external transmitter via an inductive link. The dome-shaped LCP lid encloses the circuit mounted on the LCP substrate to encapsulate the electronics in an aqueous in vivo environment. As shown in Figure 1b, the LCP-based microelectrode array used for precisely targeting the deep brain region has eight stimulation channels arranged linearly along the shank. Each channel is a rectangle of 50 μm × 160 μm. The electrode shank is designed to be 8 mm long, 300 μm wide, and 250 μm thick for targeting the ventral posterolateral nucleus (VPL) of the SD rat and located between 4.8 mm and 6.6 mm below the cortical surface [27,28]. The 250 μm thickness represents a compromise that balances the structural rigidity to penetrate the brain tissue and its desired flexibility to minimize post-implantation immune response. The arrangement of the stimulation channels ensures that the electrode array covers at least half of the VPL region of the SD rat. Each channel has a height of 50 μm and interchannel spacing of 100 μm to accommodate all eight channels within a 1 mm length.

### 2.2. Device Fabrication

#### 2.2.1. Microfabrication of an Electrode on an LCP Substrate

**Figure 2:**
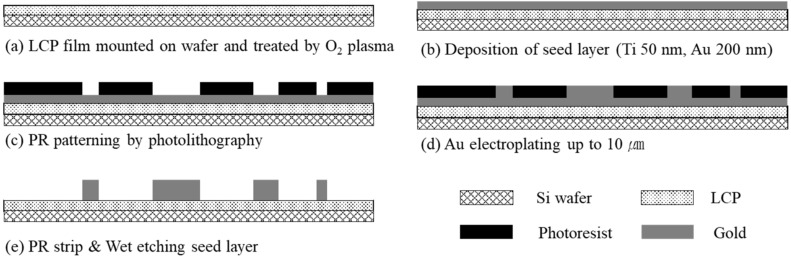
Microfabrication process for patterning electrodes on LCP. (**a**) Surface treatment using O_2_ plasma on bare LCP film mounted on a silicon wafer; (**b**) deposition of seed layers of 50 nm Ti and 200 nm Au using e-beam evaporation; (**c**) photolithography for patterning PR (AZ4620) on the seed layer; (**d**) electroplating of gold up to 10 μm; (**e**) stripping PR and wet etching metal seed layer.

The microfabrication process of the LCP-based electrode array is illustrated in Figure 2. The first step was to fix an LCP film (Vecstar CTZ-25, Kuraray, Japan) onto a 4-inch silicon wafer coated with a silicone elastomer. The surface of the LCP film was treated with oxygen plasma using a reactive ion etcher (RIE 80 plus, Oxford Instruments, Abingdon, UK) under O_2_ 100 sccm, 0.1 mTorr, and 150 W for 3 min to improve the adhesion between the film and the metal layer. Next, metal seed layers of 50 nm thick Ti and 200 nm thick Au were sequentially deposited on the film using an e-beam evaporator. Titanium served as an adhesion layer between the LCP film and the gold layer. After spin-coating the photoresist (AZ4620, Clariant Corporation, Somerville, NJ, USA), photolithography was performed to transfer the patterns on the photomask to the metal layer using a UV aligner (MA6/BA6, SUSS MicroTec, Garching, Germany). Then, gold was electroplated, creating a gold layer up to 10 μm thick, using the patterned PR as a mold, followed by a PR strip. To isolate each thick gold pattern, the seed layer was removed via two-step wet etching: gold layer removal using aqua regia (HCl/HNO_3_ = 3:1) and titanium layer with diluted HF solution (<3%).

**Figure 3:**
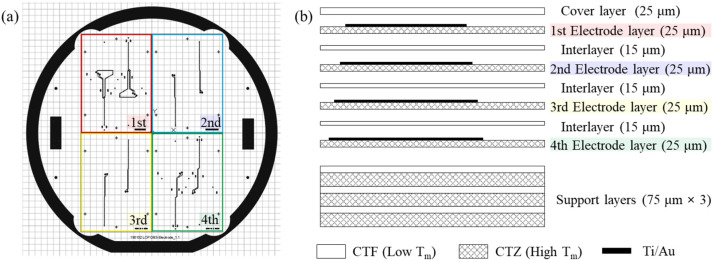
A schematic diagram of the multilayer electrode stack for optimized shank size. (**a**) The mask pattern of the electrode with four layers in each quadrant corresponds to the 1st–4th layers as color-coded in the constitution of the multilayered shank substrate in (**b**). The stack consists of four electrode layers, three support layers, six interlayers, and a cover layer.

The gold-patterned LCP, once separated from the carrier wafer, was then divided into four pieces using a UV laser. The pieces were stacked and laminated to construct a multilayered electrode shank, as illustrated in Figure 3. The eight-channel electrode array was arranged into two channels per layer, considering the constrained width of the shank (300 μm). Each layer contained alignment holes for stacking and aligning keys for laser machining for site opening and thinning. Subsequently, these pieces were combined into a 395 μm thick stack by incorporating additional layers, including a cover layer, interlayers, and supporting layers, as illustrated in Figure 3b. Additional layers were prepared from bare LCP films with high (335 °C; Vecstar CTZ series, Kuraray, Tokyo, Japan) or low (280 °C; CTF series, Kuraray, Tokyo, Japan) melting temperatures ™.

**Figure 4:**
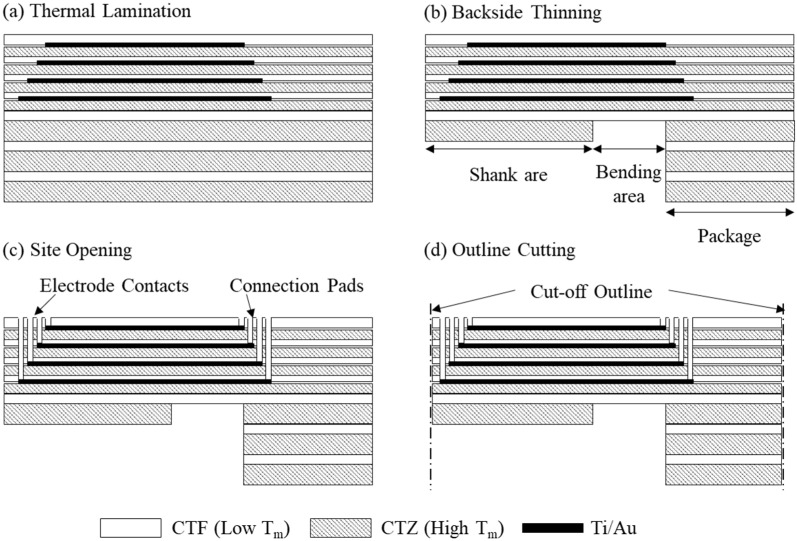
Multilayered lamination and laser machining process for electrode fabrication. (**a**) Thermal lamination of the multilayered electrode stack; (**b**) backside laser thinning of the electrode shank and bending area; (**c**) site opening of the electrode contacts and connection pads; (**d**) outlining of the electrode using UV laser.

The stack in Figure 3b was thermally bonded to construct a multilayered shank substrate via a thermal bonding process using a heating press (CH4386, Carver Inc., Wabash, IN, USA), as shown in Figure 4a. Thermal pressing was performed at 286 °C with a load of 20 kgf applied for 30 min, using LCP films with lower melting temperatures as adhesive layers. The mechanical characteristics of the electrode shank were optimized to ensure stable insertion into deep brain regions while maintaining sufficient flexibility to minimize potential tissue damage, achieved by tuning the thickness of the shank. The multilayered shank substrate was subjected to two-step backside UV laser thinning: the first step involves laser thinning of the shank area, achieving a thickness of 250 μm, while the second step focuses on localized thinning of the bending area, which needs to be flexible during the implantation procedure (Figure 4b). Subsequently, laser ablation was used to expose the electrode contacts for tissue interfacing and connection pads for interconnection with the circuit board (Figure 4c). Finally, an outline of the electrode was defined using UV laser machining to complete the electrode fabrication process (Figure 4d).

#### 2.2.2. Circuit Design

**Figure 5:**
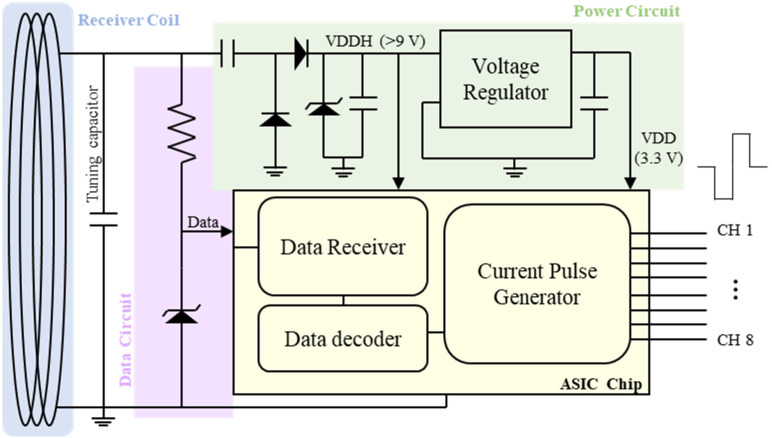
Block diagram of the stimulator circuit for the head-mountable DBS system for rats.

The wirelessly powered circuit for generating current pulses comprised a receiver coil, a power circuit, a data circuit, and a pulse-generating application-specific integrated circuit (ASIC) (Figure 5). The receiver coil received RF signals from the external coil via an inductive link. The ASIC was controlled by digital signals encoded via pulse-width modulation (PWM) over a carrier frequency of 2.54 MHz. A 23-turn receiver coil with a diameter of 11 mm was implemented on a PCB. The power circuit rectified and regulated the received signal to produce two levels of DC voltages: 3.3 V (VDD) for operating the logic circuit and a high compliance voltage (VDDH, typically over 9 V) for the analog output of the ASIC to secure robust current injection through the neural interface. The data circuit rectified the inbound PWM signal into a half-wave signal fed into the ASIC. The 16-channel current stimulator ASIC chip, previously published in [29], decoded the received PWM signal to generate biphasic current pulses for each channel with programmed pulse parameters. The parameters of the stimulation pulses were programmable in terms of the duration (from 0 to 630 μs in 10 μs steps), pulse rate (from 20 to 230 Hz in 5 Hz steps), and amplitude (from 0 to 10.23 mA in 10 μA steps).

#### 2.2.3. Integration

**Figure 6:**
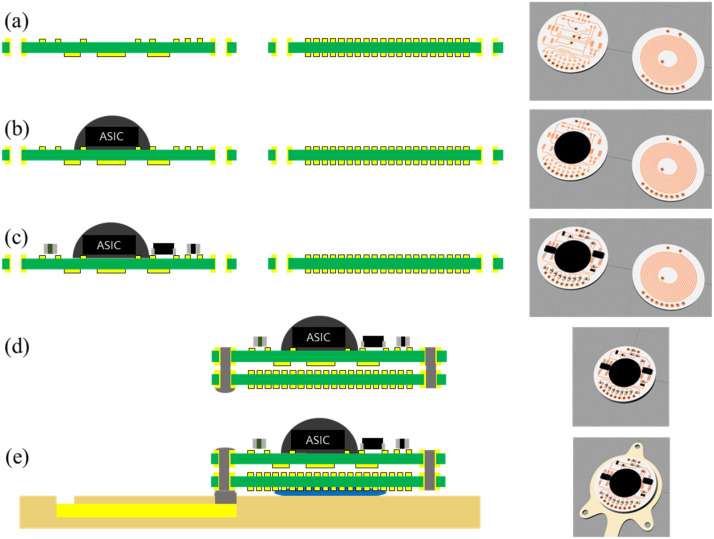
Integration steps for circuit PCB, coil PCB, and LCP electrode array assembly. (**a**) Preparation of the circuit PCB (left) and the coil PCB (right); (**b**) wire-bonding the current stimulation ASIC; (**c**) soldering discrete elements, including a regulator, diodes, resistors, and capacitors; (**d**) stacking and interconnection of circuit and coil boards; (**e**) final assembly and interconnection of LCP electrode array.

The stimulator circuit was implemented on a circular FR-4 printed PCB with an 11 mm diameter and a 0.4 mm thickness. The current stimulator ASIC was wire-bonded in the center of the board, surrounded by a 3.3 V regulator (TPS76333, Texas Instrument, Dallas, TX, USA), two Schottky diodes (BAT30SWFILM, STMicroelectronics, Geneva, Switzerland), two Zener diodes (GDZ18LP3 and GDZ3V3LP3, Diodes Inc., Plano, TX, USA), DC-block capacitors, and other SMD resistors and capacitors, along with connection vias to the electrode array and the coil (Figure 6a–c). A double-layered planar spiral receiver coil fabricated on a separate PCB of the same size and materials as the circuit had an outer diameter of 11 mm, an inner diameter of 4 mm, and 11.5 turns per layer. The circuit components were mounted only on the upper side of the PCB to minimize the overall thickness of the circuit during the assembly.

Figure 6d,e show the integration process of the circuit PCB, the coil PCB, and the LCP electrode array. The circuit and coil boards were interconnected by soldering two through-hole vias. Nine vias (eight channels and one reference) on the circuit PCB were connected to the contact pads of the LCP electrode array by filling the holes with silver epoxy (H20E; Epoxy Technology, Billerica, MA, USA). Subsequently, epoxy glue was applied to the space between the assembled circuit PCB and the LCP electrode array. The entire assembly was then cured on a hot plate at 110 °C for 30 min, ensuring the robust and stable integration of the components within the device.

#### 2.2.4. Packaging

In contrast to the conventional thermal lamination used for flat LCP films, the packaging of active electronic components introduces the unique challenge of bonding nonplanar LCP structures without affecting electronic functionality. This study used a specialized packaging technique known as the spot-welding process, previously reported in [30], which involves applying localized heat and pressure to bond nonplanar LCP layers. The spot-welding technique allows for localized bonding around the perimeter of the circular package while ensuring that the electronic components, such as the ASIC chip and SMD components, are unaffected by heat and pressure.

**Figure 7:**
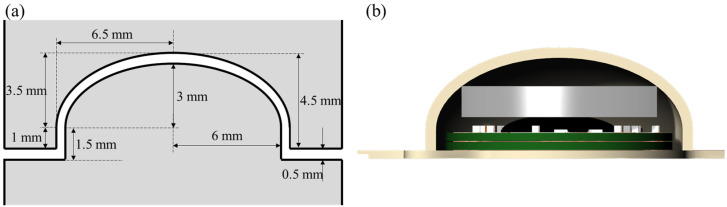
Cross-sectional illustration of the packaging process of the stimulator circuit. (**a**) Geometries of the metal jig for the thermal deformation of the LCP film to create a dome-shaped package lid and (**b**) the inside of the package encasing a magnetic, circuit PCB and a coil PCB.

The first step in packaging is preparing a 5 mm high, 500 μm thick dome-shaped package lid with an outer diameter of 13 mm, as illustrated in Figure 7a, to enclose the circuit components. To create a dome-shaped LCP lid, a pair of aluminum jigs were used to thermally deform the flat LCP films, as shown in Figure 7b. Five layers of alternating 100 μm thick CTZ (high *T_m_*) and CTF films (low *T_m_*) were laminated, followed by thermal deformation at 285 °C with 100 kgf load for 30 min within the metal jig. Each lid was equipped with a coin-type neodymium magnet, 8 mm in diameter and 1 mm in thickness, attached at the center to align with the external coil through the scalp to achieve optimal power transfer efficiency. Finally, after mounting the LCP lid onto the LCP substrate assembled with the circuit and coil, the perimeter of the lid was bonded to the substrate via a spot-welding process involving the localized application of heat and pressure.

### 2.3. Evaluation

#### 2.3.1. FEM Simulation

**Figure 8:**
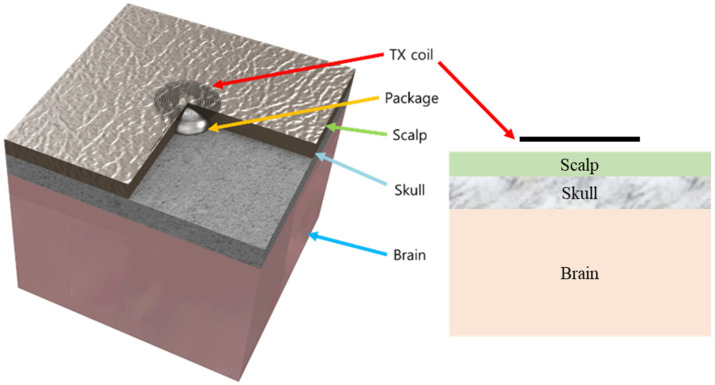
The 3D brain–skull–scalp model used in the FEM simulation for SAR analysis.

To assess the RF radiation safety of the proposed system, the specific absorption rate (SAR) was computed using an FEM simulation with a human brain–skull–scalp model in ANSYS HFSS (ANSYS Inc., Canonsburg, PA, USA), as shown in Figure 8. The SAR quantifies the amount of energy absorbed by living tissues when exposed to electromagnetic fields. It is defined as the absorbed power over the tissue mass in watts per kilogram (W/kg) and can be expressed using the following formula [31]:

(1) $$SAR=\frac{1}{V}\int \frac{\sigma \left(r\right){\left|E\left(r\right)\right|}^{2}}{\rho (r)}dr\;\left[W/\mathrm{kg}\right]\;\left(\mathrm{for}\;100\;\mathrm{kHz}∼10\;\mathrm{GHz}\right),$$

where σ is the electric conductivity in Siemens per meter (S/m), E is the root mean square of the electric field in volts per meter (V/m), and ρ is the density of the sample in kilograms per cubic meter (kg/m^3^).

In Figure 8, the human brain model demonstrates the placement of the proposed LCP stimulator package on top of the skull layer, while the external transmitter is encapsulated by a 2 mm thick silicone elastomer and positioned 1 mm above the scalp surface. This model measures 60 mm × 60 mm and includes the scalp, skull, brain, and LCP layers, each with varying thicknesses and electromagnetic parameters, as summarized in Table 1. In particular, the parameters for the skull and scalp are representative of the parietal region of the human head, which is the target location of the system. For the SAR simulation, sinusoidal waves with a frequency of 2.54 MHz and an amplitude of 20 V were applied to the TX coil, assuming the maximum energy that can be delivered via the TX coil during actual system operation.

#### 2.3.2. In Vivo Experiment

**Figure 9:**
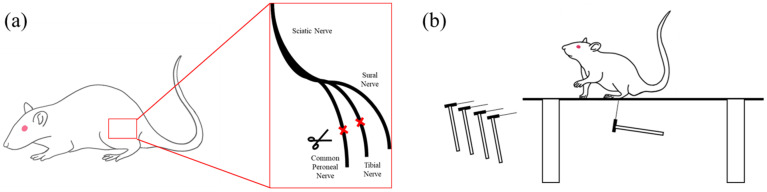
Methods of in vivo experiment in a rat model of neuropathic pain. (**a**) Schematic illustration of spared nerve injury (SNI) pain modeling procedures. The common peroneal and tibial nerves were ligated using a 5–0 silk suture, and 2–3 mm of the nerves were removed (marked in red). (**b**) von Frey filament test.

To evaluate the efficacy of the proposed DBS system, we conducted an in vivo behavioral test on a rat model of neuropathic pain using the spared nerve injury (SNI) method, which is a reliable and responsive pain model [2,3,36]. The SNI modeling procedure is shown in Figure 9a. The rat was anesthetized, and the three branches of the sciatic nerve were exposed. After they were exposed, the common peroneal and tibial nerves were tightly ligated with a non-absorbable 5.0 silk suture and sectioned distal to the ligation, removing 2–4 mm of the distal nerve stump. The sural nerve remained intact, and the muscle and skin were closed into two layers. The animals were maintained for two weeks after surgery to induce chronic pain. After modeling, the pain threshold on the lateral side of the sole of the modeled limb decreased. To assess the pain levels in the rats, we performed the von Frey filament test, as shown in Figure 9b. This test measures the pain response scores after thin filaments of various strengths are applied to the pain-modeled region. We compared the pain thresholds of rats with and without DBS stimulation and confirmed the effect of stimulation on these thresholds. The waveform and target tissues of the DBS stimulus were determined on the basis of a previous study [28]. We varied the stimulation amplitudes at 130 Hz and 60 μs and observed the difference in the thresholds for each amplitude.

**Figure 10:**
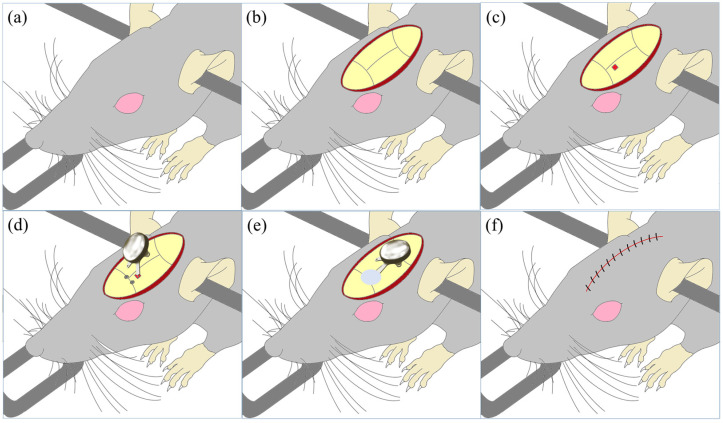
LCP-DBS implantation process in a rat. (**a**) Anesthetizing and securing the rat in a stereotaxic instrument; (**b**) making an incision on the scalp; (**c**) locating the target region on the basis of a brain atlas and drilling a hole in the skull; (**d**) inserting the electrode to a predefined depth and fixing it with dental resin; (**e**) bending the electrode and securing the package with a surgical screw; (**f**) suturing the incision.

Because the proposed implantable device is monolithic, it differs from conventional modular systems. The monolithic system may have advantages in terms of package reliability or miniaturization, but it requires a different implantation method from the existing systems. Thus, we designed the implant method for the in vivo experiments to verify this system, as shown in Figure 10.

An incision was made on the scalp near the vertex after anesthetizing and fixing the subject to a stereotaxic surgical instrument. After the hemostasis of the bleeding from the exposed subcutaneous tissue and skull area, the location of the VPL was marked, and the skull was drilled. The implantable device was vertically fixed with a custom-made holder for the instrument, and the electrode was inserted into the VPL area. After the electrode was fixed to the skull using dental cement, the bending zone between the electrode and the package was bent; the package was placed above the skull. It was fixed to the skull using surgical screws inserted into the holes around it, and the incision was sutured.

## 3. Results

### 3.1. Fabrication of an Electrode Array and a System Substrate

**Figure 11:**
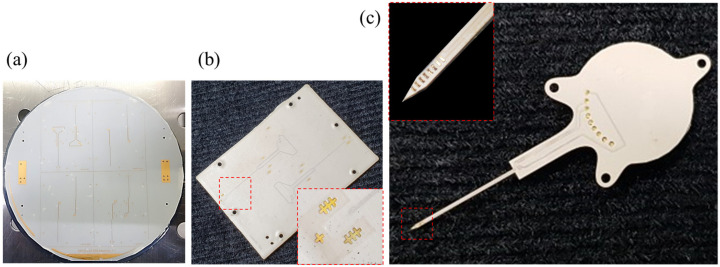
Fabrication results of LCP-based multilayered DBS electrode array. (**a**) The 4-inch LCP layer after microfabrication process showing gold patterns in each quadrant to be cut and stacked; (**b**) the multilayered LCP substrate after thermal lamination (inset: align keys around the electrode contacts); (**c**) completed system substrate after laser machining for site opening, backside thinning and outlining, and hosting with an eight-channel electrode array and connection pads for interconnection to circuit PCB (inset: closer view of the electrode contacts).

Figure 11 shows the results of the microfabrication process on a 4-inch scale and the subsequent multilayered lamination with laser machining steps, producing the LCP substrate for the system. In Figure 11a, each quadrant hosts two electrode channels, which are subsequently cut and laminated with additional interlayers, supporting layers, and a cover layer to create a 395 μm thick eight-channel system substrate, as presented in Figure 11b. The gold electrode patterns are insulated by the LCP cover layer with the alignment keys exposed, facilitating the subsequent laser machining steps, including site opening, backside thinning, and outlining, as shown in Figure 11c. The system substrate contains eight microelectrodes located near the tip of the shank and eight connection pads for interconnecting with the circuit PCB. The shank of the electrode is 260 μm thick and 300 μm wide, ensuring stable insertion into the deep brain region while maintaining sufficient flexibility.

**Figure 12:**
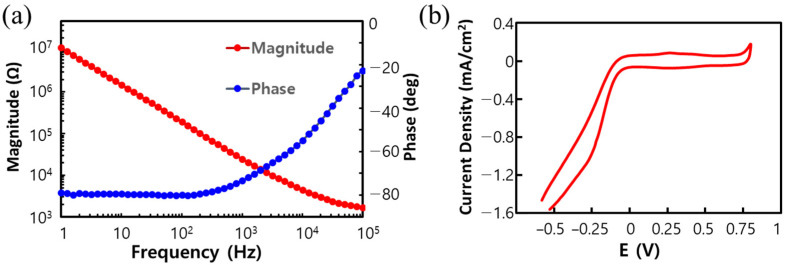
Result of electrochemical analysis of the fabricated DBS electrodes: (**a**) EIS and (**b**) CV.

The electrochemical properties of the fabricated electrode array were evaluated via electrochemical impedance spectroscopy (EIS) and cyclic voltammetry (CV) using an impedance analyzer (Solartron 1260/1287, AMETEK Inc., Berwyn, PA, USA) with a three-electrode configuration. Figure 12 shows the measured EIS and CV data for the electrodes. The impedance magnitude at 1 kHz was 24.55 kΩ, with a phase angle of −73.28°, and the cathodic charge storage capacity calculated from the CV curve was 0.549 mC/cm^2^. These values fall within the typical range for an electroplated gold surface with an opening size of 50 μm × 160 μm.

**Figure 13:**
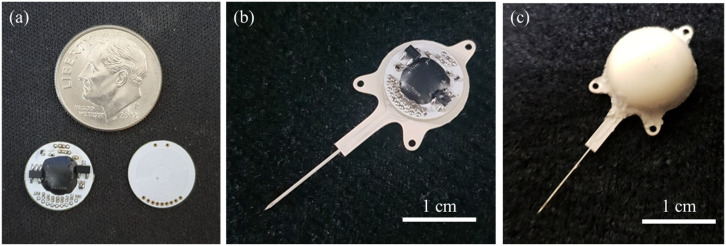
Results of the packaging process. (**a**) A circuit PCB and a coil PCB; (**b**) after assembly onto the LCP system substrate with an electrode array; (**c**) package lid bonded by spot welding.

Figure 13 presents the step-by-step integration and packaging processes. The circuit and coil PCB shown in Figure 13a were stacked and soldered together for mounting on the LCP system substrate, which carried the electrode array, as shown in Figure 13b. The channels on the system substrate were interconnected by filling the via with Ag epoxy. Finally, the LCP package lid encasing the circuit, the coil, and the magnet was thermally bonded to the LCP substrate via spot welding, as shown in Figure 13c.

### 3.2. Evaluation

#### 3.2.1. SAR Safety Verification

**Figure 14:**
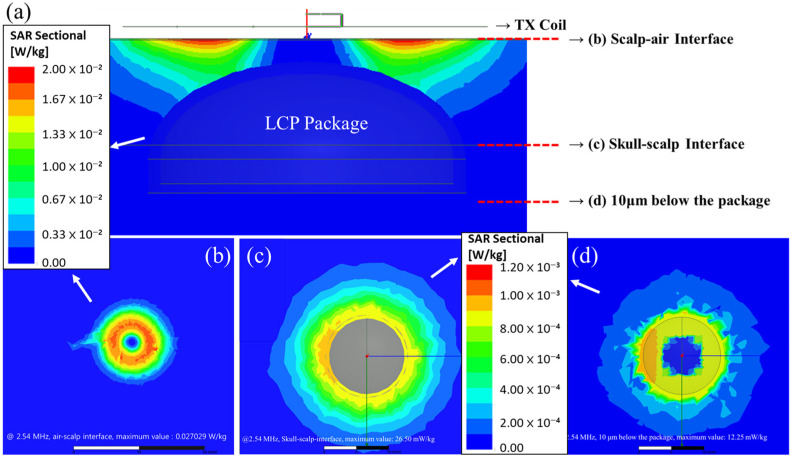
FEM simulation results for assessing the SAR upon the wireless powering of the proposed stimulator device. (**a**) Cross-sectional view around the implanted package and the transmitter coil; the top-view distribution of the SAR (**b**) at the air–scalp interface, (**c**) at the scalp–skull interface, and (**d**) 10 μm underneath the package (corresponding scale bars are marked by arrows).

The FEM simulation results for estimating the SAR during wireless powering of the implanted device using a TX coil are presented in Figure 14. These plots present the SAR distributions in the cross-sectional (sagittal) and transverse planes at varying depths from the skin. Notably, the SAR was most concentrated near the TX coil and gradually decreased with increasing distance from the coil. The maximum SAR values were observed at the air–scalp interface, yielding 27.45 mW/kg. Importantly, this value is well below the permissible threshold of 80 mW/kg for the SAR, indicating the system’s safety in terms of RF radiation [37].

#### 3.2.2. In Vivo Animal Experiment

**Figure 15:**
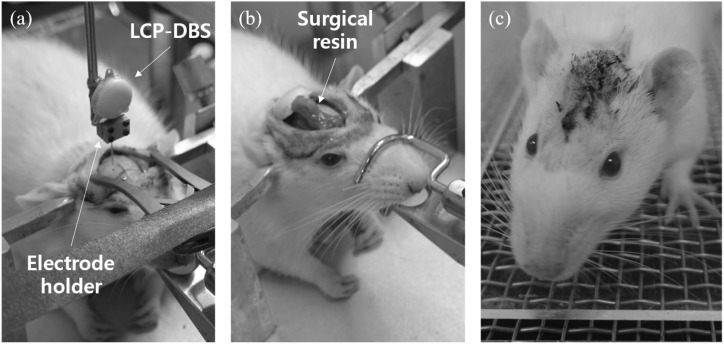
Implantation process of the LCP DBS. (**a**) After anesthetization, incision, and positioning of the device above the skull targeting VPL; (**b**) after insertion of the electrode and fixing of the package; (**c**) after a week of recovery postsurgery.

Figure 15 shows the implantation process of the DBS system implantable device. As shown in Figure 15a, a custom-designed electrode holder was used to fix the LCP-DBS electrode during stereotaxic surgery. The holder had four screw holes to secure the frame, body, and rail in the middle to prevent misalignment. The electrode had a bending zone to facilitate loading, which required full coverage by the holder during implantation. Figure 15c shows a rat undergoing behavioral testing after the device was fully implanted and acclimated. The rats moved freely after implantation, and no sutures were torn nor were inflammatory reactions observed until the subjects were sacrificed.

**Figure 16:**
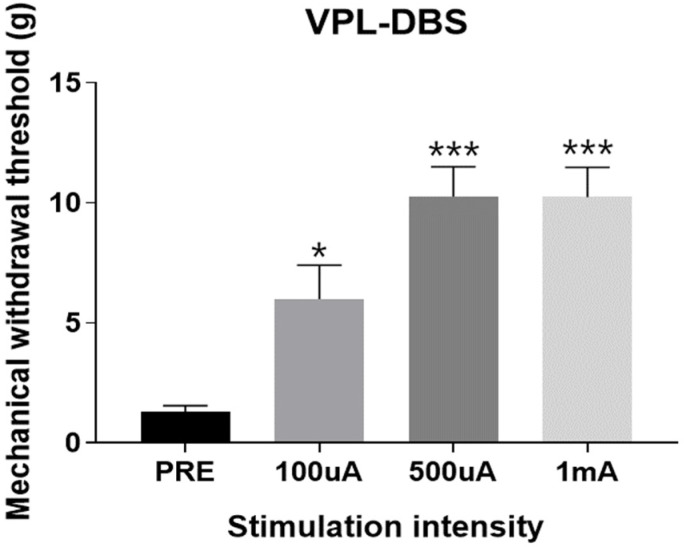
Result of in vivo behavioral test using a von Frey filament in a rat model of neuropathic pain. The increase in the mechanical withdrawal threshold in response to changes in the microelectrical stimulation intensity. The bars indicate the mean ± SEM. One-way ANOVA with Dunnett’s multiple-comparison post hoc test; * *p* < 0.05, *** *p* < 0.001.

Figure 16 shows the results of in vivo behavioral tests on the SD rat neuropathic pain model. The horizontal axis represents the stimulation intensity, and the vertical axis represents the withdrawal threshold, which is the strength at which the rat feels pain and lifts its feet. The results show that before the DBS was applied (PRE in the graph), the withdrawal threshold was 1.297 ± 0.2546 g. However, when a current stimulation of 100 μA was applied, the threshold increased up to 5.996 ± 1.413 g (* *p* < 0.05). When a current stimulation of 500 μA was applied, the threshold rose to 10.27 ± 1.236 g (*** *p* < 0.001). When 1 mA amplitude was applied, the threshold was similar to that on stimulation with a current of 500 μA (10.24 ± 1.236 g; *** *p* < 0.001). Considering that the stimulation method applied in the experiment was a bipolar stimulus, a 16 V compliance voltage was applied. Although this circuit has a compliance voltage of up to 18 V, the inductive link is less efficient than that in the benchmark test when the device is implanted in the body of the animal. Therefore, the saturated withdrawal threshold was induced via the saturation of the current pulses from the implantable device owing to the high electrochemical impedance of the electrode sites.

## 4. Discussion

This study presents a fully implantable and miniaturized DBS device for rats using biocompatible LCP films as the substrate and the packaging material. LCP offers several advantages. For example, it enables compact and lightweight miniaturization; ensures minimal moisture absorption; and allows for RF transparency, chemical stability, mechanical robustness, and compatibility with standard microfabrication processes. The lower moisture absorption rate (<0.04%) of LCP compared to those of conventional biocompatible polymers, such as polyimide (~2.8%) and parylene-C (0.06–0.6%), is the most advantageous property of LCP as a substrate and the packaging material for implantable neuroprosthetic devices. The LCP material is also attracting increasing attention for optical applications [38]. Additionally, LCP enables the monolithic integration of the system, in which the electrode array, the circuit, and the receiver coil are all packaged within the same LCP material used as the substrate. This eliminates the need for feedthroughs commonly found in conventional metallic packages that are susceptible to water leakage.

The key characteristic of the LCP electrode is its thin profile, which measures only one-tenth of the thickness of conventional DBS electrodes. This unique design achieves a delicate balance between rigidity and flexibility. The electrode possesses sufficient stiffness to penetrate the brain tissue without the need for a mechanical cannula yet retains the flexibility to minimize tissue damage during and after implantation. The stimulator circuit is wirelessly powered and controlled by an external device placed outside the body, contributing to the battery-free miniaturization of the package. This enables the device to be mounted on the head of rats, which is much smaller than that for humans, in contrast to commercial DBS systems with battery-powered IPGs implanted in the chest and connected by subcutaneous lead wires. Such monolithic head-mountable devices can eliminate the need for replacement surgery and significantly reduce the risk of infection and discomfort in patients.

It is to be noted that no vias have been implemented in the LCP layers in this study, primarily because it was not necessary, given the electrode structure, the number of channels, and the interconnection scheme; however, the LCP electrode can be embedded by interlayer connection through vias created by electroplating or epoxy filling [39]. Also, gold was used as an electrode material, but the proposed electrode technology is also compatible with typical nanomaterials, such as iridium oxide and graphene, for enhanced electrochemical performance [24,40,41]. The total thickness (395 μm) of the proposed array is a result of the optimization of rigidity and flexibility. The mechanical properties of LCP as a depth probe have been previously investigated [42], in which the proper design of the LCP structure could ensure that the electrode was rigid enough to penetrate the cortex yet had high flexibility. These characteristics enable the implantation of electrodes into the cortex without the use of additional tools, such as dissolvable coatings, metallic stiffeners, or cannulas, which ultimately simplifies surgical procedures and prevents post-operative tissue damage.

Several issues must be addressed to successfully translate the proposed system into the clinical phase. First, the current microfabrication process for an electrode array based on a 4-inch wafer must be migrated to larger wafers, at least 8 inches in diameter, to accommodate the electrode design required for human models. Second, incorporating a recording functionality for real-time monitoring of brain signals during implantation surgery is expected to ensure precise electrode placement. This enhancement can be achieved by integrating a recording circuit, back-telemetry logic, and additional electrode channels into the device using a circuit design and a microfabrication process similar to those presented in this study.

## Figures and Tables

**Table 1:** Parameters of FEM analysis for the SAR model.

Tissue	Thickness (mm)	Conductivity (S/m)	Relative Permittivity	Relative Permeability
Scalp	4.284 [32]	0.0512 [33]	795 [33]	1 [34]
Skull	5.915 [35]	0.0304 [33]	92.4 [33]	
stadBrain	40	0.220 [33]	855 [33]	
LCP	0.5	10^−17^	3.3	1

## Data Availability

The data that support the findings of this study are available upon request from the corresponding author.
